# Interactions between immunoglobulin evasion systems in *Mycoplasma gallisepticum* and their impact on antibody-dependent complement-mediated killing

**DOI:** 10.1128/iai.00373-26

**Published:** 2026-10-12

**Authors:** Dilhani Ekanayake, Anna Kanci Condello, Nadeeka K. Wawegama, Glenn F. Browning, Kelly A. Tivendale

**Affiliations:** 1 Asia-Pacific Centre for Animal Health, Melbourne Veterinary School, Faculty of Science, The University of Melbourne 2281, Melbourne, Victoria, Australia; University of California Davis, Davis, California, USA

**Keywords:** *Mycoplasma gallisepticum*, Protein-M, CysP, MIB-MIP, antibody, immune evasion, protease, virulence, complement-mediated killing

## Abstract

*Mycoplasma gallisepticum* (MG) causes chronic respiratory disease, which is thought to be contributed to by its capacity to evade the immune response of infected birds. It possesses multiple copies of the genes encoding the mycoplasma immunoglobulin binding-protease (MIB-MIP) system, which binds and cleaves avian immunoglobulins (Igs), but also encodes homologs of the Ig-binding Protein-M of *M. genitalium* and *M. pneumoniae* and the Ig-cleaving CysP of *M. synoviae*. However, the interactions between these homologs, their host-specificity, and their interplay with the MIB-MIP system have not been defined. Here, the putative Protein-M and CysP in MG were expressed as recombinant glutathione-S-transferase (GST)-fusion proteins, functionally characterized, and their interactions with different MIB-MIP system components, as well as their impact on antibody-dependent complement-mediated killing of MG, examined. GST-Protein-M bound avian and mammalian immunoglobulins, but did not interact with the MIP proteases. GST-CysP selectively cleaved free chicken IgY and IgY complexed with GST-MIB or GST-Protein-M. GST-Protein-M, GST-CysP, and GST-MIB-MIP proteins all bound ATP, but only GST-CysP had ATPase activity. Sera from MG-infected chickens contained antibodies against a subset of MIP proteins, but not antibodies against Protein-M or CysP. The capacity of rabbit anti-MG antibodies to enhance complement-mediated killing of MG was inhibited by GST-MIB-MIP and GST-Protein-M. GST-CysP could not cleave rabbit IgG and had no detectable effect in the antibody-dependent complement-mediated killing assays. Collectively, these findings establish that Protein-M and CysP function as additional Ig-targeting systems in *M. gallisepticum* alongside the MIB-MIP system through distinct but complementary mechanisms, enhancing immune evasion and persistence of this important pathogen.

## INTRODUCTION

Mycoplasmas have a streamlined genome and, as a result, lack a cell wall and many of the genes responsible for metabolism in other bacteria. These adaptations make them dependent on their host for survival (1, 2). However, they cause chronic diseases, persisting on mucosal surfaces despite their hosts’ immune responses (3, 4). *Mycoplasma gallisepticum* (MG), the pathogen of focus in this study, causes chronic respiratory disease in chickens and infectious sinusitis in turkeys, diseases with significant clinical and economic implications (5, 6). Following infection, the host’s humoral immune response typically begins with an increase in concentrations of mycoplasma-specific IgM during the acute phase, with concentrations of mycoplasma-specific IgG and IgA increasing in the chronic phase (7–10). Despite this response, some mycoplasmas can persist within their hosts, a capacity attributed to phase variation in their cell-surface antigens, as well as other immune evasion strategies (11, 12). Many mycoplasma species secrete or surface-express proteases that can cleave and inactivate host immunoglobulins (11, 12). The ureaplasmas, a phylogenetic group within the mycoplasmas, secrete an IgA1 protease that cleaves the hinge region between the Fab and Fc domains, disrupting Fc-mediated complement activation and opsonization, enabling them to escape antibody-mediated phagocytosis (11, 13–16). Similarly, in *M. synoviae*, a pathogen of poultry, a cysteine protease, CysP, cleaves chicken IgY (the equivalent of mammalian IgG) into Fab and Fc fragments (17). A homolog of the *cysP* gene is found in the distantly related poultry pathogen *M. gallisepticum*, and, like a number of virulence-associated genes in this species, was probably acquired via horizontal gene transfer.

In addition to immunoglobulin proteases, many bacteria, including gram-positive pathogens, express immunoglobulin-binding proteins (IBPs) that contribute to evasion of host defenses (18, 19). IBPs like Protein A (*Staphylococcus aureus*), Protein G (group C *Streptococcus* sp.), and M-protein (group A *Streptococcus* sp.) bind the Fc region of immunoglobulins (18, 20–22), thereby subverting antibody-mediated effector responses such as complement-mediated killing and phagocytosis. Immunoglobulin-binding proteins have also been found in mycoplasmas (11, 23). The human pathogen, *M. penetrans*, encodes an IgA-specific membrane lipoprotein (24), while *M. genitalium* and *M. pneumoniae* encode Protein-M, a transmembrane protein that binds to the Fab region of both human and non-human IgG light chains (12, 23). Protein-M preferentially binds free IgG, targeting the V_L_ domain of human IgG, thereby blocking antigen-antibody binding (12, 23). It also binds human IgA and IgM, and thus is likely to interfere with multiple stages of the humoral immune response (18). Homologs of Protein-M have been identified in *M. pneumoniae*, *M. penetrans, M. iowae*, and *M. gallisepticum*, all of which belong to the *pneumoniae* phylogenetic group (12, 23).

Many mycoplasmas express a two-protein, mycoplasma immunoglobulin binding and mycoplasma immunoglobulin protease (MIB-MIP) system (23, 25), which has recently been shown to be complemented by an F_0_F_1_-type ATPase. This system is absent in *M. pneumoniae* and *M. genitalium*, which instead express the IgG-binding Protein-M (23, 25). However, *M. gallisepticum* encodes homologs of all three systems, Protein-M, CysP, and the MIB-MIP system, suggesting that they may make overlapping or synergistic contributions to immune evasion. Our previous work characterized the function of the proteins encoded by the multiple homologs of the MIB-MIP system genes in *M. gallisepticum* and their involvement in evasion from the avian immune response (26). Building on this, the study described here aimed to functionally characterize the Protein-M and CysP of *M. gallisepticum*, and to evaluate their potential interplay with the MIB-MIP system. Additionally, we investigated the immunoreactivity of these proteins, their capacity to bind ATP, and their potential role in modulating antibody-dependent complement-mediated killing of *M. gallisepticum*.

## RESULTS

### Protein-M and CysP of *M. gallisepticum*

**Fig 1:**
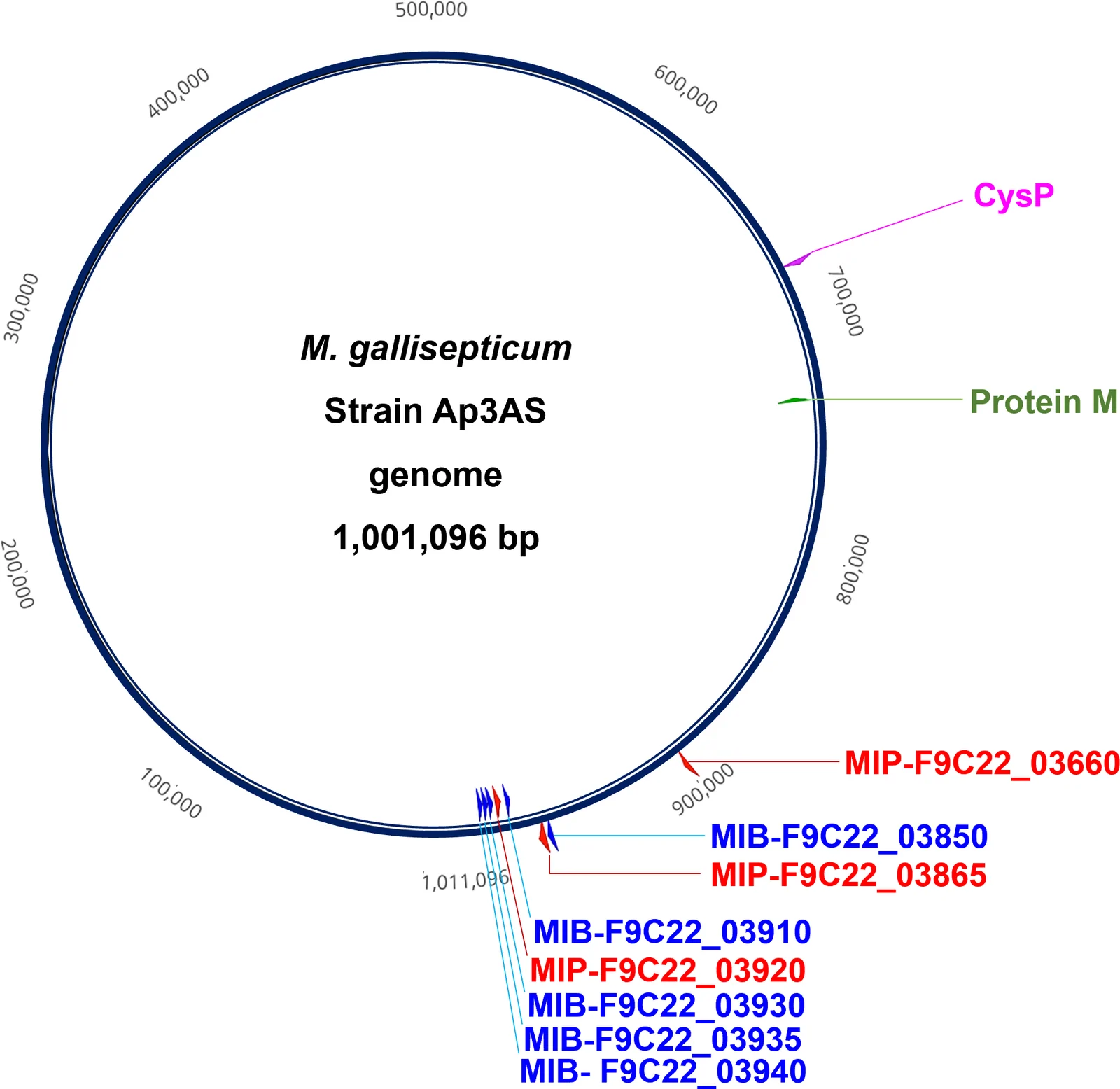
Schematic representation of the *M. gallisepticum* Ap3AS genome (GenBank: CP044624.1), highlighting genes encoding homologs of Protein-M, CysP, and members of the MIB-MIP system. Protein M (green arrowhead) and CysP (pink arrowhead) genes are located at distinct loci, spatially separated from the genes encoding MIB (blue arrowheads) and MIP (red arrowheads) homologs.

Genomic analyses showed that the putative Protein-M homolog F9C22_03005 (GenBank: UQZ95710.1) and CysP homolog F9C22_02750 (GenBank: UQZ95669.1) were located distantly from each other in the genome of *M. gallisepticum* strain Ap3AS (GenBank: CP044624.1) and were positioned separately from the MIB-MIP gene clusters (Fig. 1). The protein sequence of the putative Protein-M was 516 amino acids in length and HHpred analysis (27, 28) predicted that it contained a leucine-rich Protein-M TD motif found in the MG_281 gene of *M. genitalium* (probability = 99.96%, *e* value = 2.6e−30). DeepTMHMM analysis (29) predicted F9C22_03005 to be a cell surface-expressed membrane protein with an N-terminal signal peptide of 32 residues. Comparative analysis of Protein-M homologs in different *M. gallisepticum* strains detected a high level of conservation, with 96–98% amino acid similarity and 95–97% identity (Table S1). BLASTX analysis identified Protein-M homologs in other avian mycoplasmas, including *M. imitans*, *M. tullyi*, *M. bradburyae*, and *M. iowae*, with 41–84% amino acid similarity between the homologs (Table S1). Protein-M in *M. gallisepticum* was quite divergent from its homologs in the human pathogens, *M. genitalium*, *M. pneumoniae*, and *M. penetrans*, with only 38–41% amino acid similarity (Table S1). Protein-M in *M. gallisepticum* also had a low level of similarity (29–35%) with all five MIB proteins in *M. gallisepticum* (Table S2).

Conserved domain analysis of CysP detected a papain-like C1 peptidase domain (interval, 54–295; *e* value = 5.23e−40) and a lectin-like superfamily domain that is found in the cysteine protease Cwp84 (interval, 307–423; *e* value = 2.49e−10). HHpred analysis also detected multiple cysteine protease hits between amino acid residues 54 and 422. CysP was 564 amino acids in length, and DeepTMHMM analysis predicted an N-terminal signal peptide (27 residues), an extracellular domain (residues: 28–543), a transmembrane helix (residues: 544–555), and a short cytoplasmic tail (residues: 556–564). CysP homologs were highly conserved across *M. gallisepticum* strains (99–100% amino acid similarity) and were all very similar to the CysP in *M. synoviae* (≥90% amino acid similarity). Homologs in other avian *Mycoplasma* species, including *M. pullorum*, *M. gallinarum*, *M. glycophilum*, *M. gallopavonis*, *M. gallinaceum*, and *M. meleagridis*, had 43–67% amino acid sequence similarity with the CysP of *M. gallisepticum* strain Ap3AS (Table S3). Limited amino acid similarity (28%) was detected between CysP and the three functional MIP proteases in *M. gallisepticum* strain Ap3AS (Table S4).

SDS-PAGE and Western blotting showed that the purified recombinant GST-Protein-M and GST-CysP had the expected molecular weights of approximately 85 and 80 kDa, respectively (Fig. S1).

### Protein-M of *M. gallisepticum* bound avian and mammalian immunoglobulins

**Fig 2:**
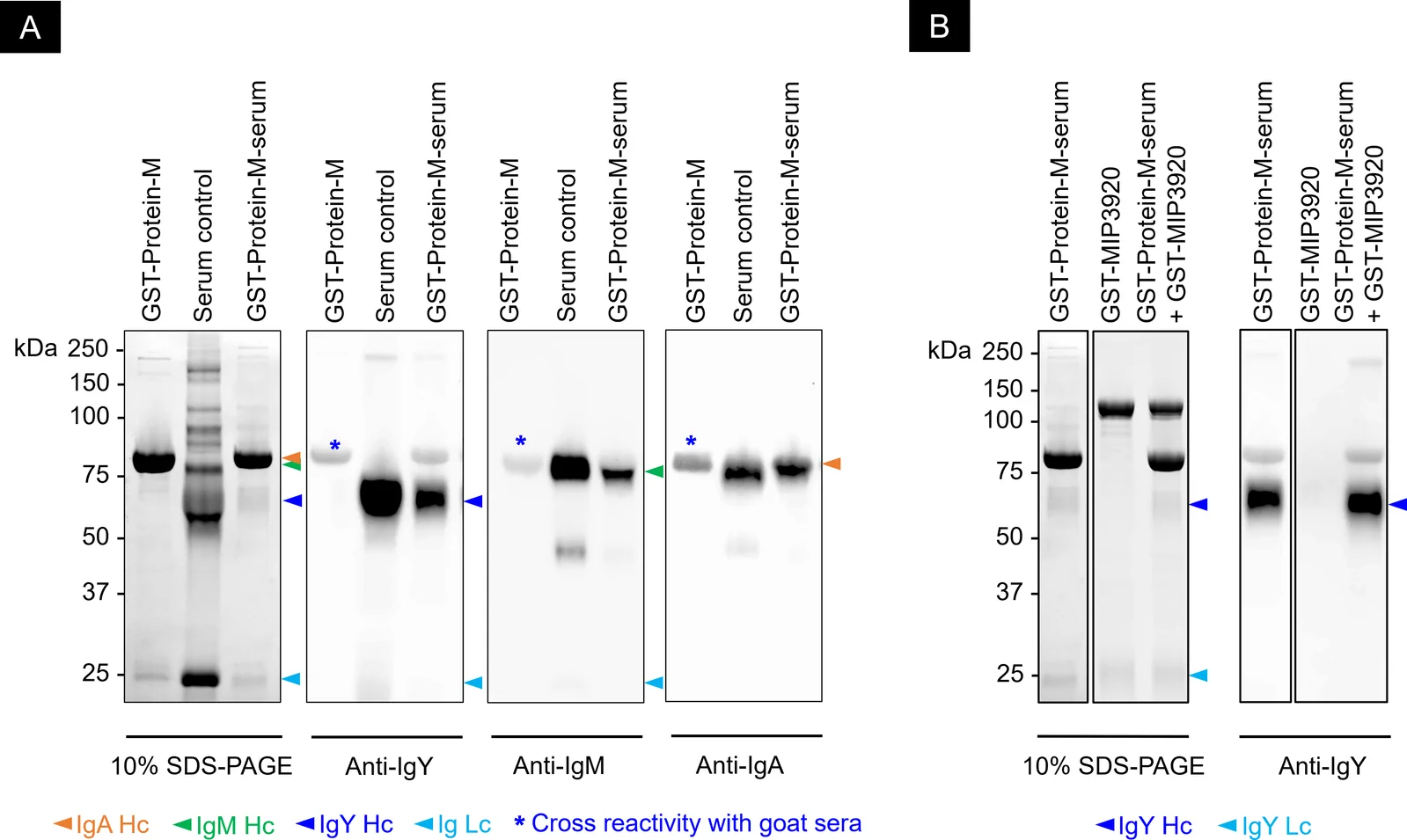
(**A**) Immunoglobulin binding capacity of recombinant Protein-M of *M. gallisepticum*. Lanes labeled GST-Protein-M-serum contain proteins eluted from glutathione columns to which a solution of GST-Protein-M and diluted chicken serum had been applied. GST-Protein-M (~85 kDa) and diluted chicken serum were included as controls. The four panels show the 10% polyacrylamide SDS-PAGE gels and Western blots probed with goat antisera against chicken IgY, IgM, or IgA. Orange arrowheads, ~75 kDa IgA heavy chains; green arrowheads, ~75 kDa IgM heavy chains; dark blue arrowheads, ~65 kDa IgY heavy chains; light blue arrowheads, ~25 kDa Ig light chains; dark blue asterisks (*), cross-reactivity of GST-Protein-M with the goat antibodies. (**B**) Interaction between recombinant Protein-M and MIP proteases of *M. gallisepticum*. Eluate from a glutathione column to which a solution of GST-Protein-M and diluted chicken serum had been applied was incubated with purified GST-MIP proteins (~112 kDa; GST-MIP3920 shown as a representative of all MIP proteins). GST-Protein-M-serum eluate alone and GST-MIP3920 alone were included as controls. Dark blue arrowheads, ~65 kDa IgY heavy chains; light blue arrowheads, ~25 kDa IgY light chains.

SDS-PAGE analysis of affinity-purified complexes following incubation of GST-Protein-M with diluted chicken serum detected two additional protein bands (~65 kDa and ~25 kDa), in addition to the expected ~85 kDa GST-Protein-M band (Fig. 2A). Western blotting with goat anti-chicken IgY (H+L) antibody confirmed that these bands were the heavy and light chains of IgY, respectively. The capacity of GST-Protein-M to bind chicken IgM and IgA was also confirmed by Western blotting (Fig. 2A). GST-Protein-M also bound the goat anti-GST antibody (Fig. 2A). Binding assays with sera from other avian species, including turkeys, ducks, and pheasants, yielded bands of similar sizes, indicating that GST-Protein-M has the capacity to bind IgY, IgM, and IgA across different avian species (Fig. S2A). Binding assays with mammalian (goat, sheep, rabbit, horse, pig, and Tammar wallaby) sera yielded distinct bands at ~65 kDa, corresponding to the IgM heavy chain, ~50–55 kDa, corresponding to the heavy chains of IgG and IgA, and ~25 kDa, corresponding to the light chains of IgM, IgG, and IgA. (Fig. S2A).

### MIP proteases did not interact with the Protein-M-IgY complex

Eluates from the GST-Protein-M immunoglobulin-binding assay were incubated with purified recombinant GST-MIP proteins to assess whether *M. gallisepticum* MIP proteases could cleave IgY bound to GST-Protein-M. SDS-PAGE and subsequent Western blotting revealed that IgY (~65 kDa) heavy chains remained intact, indicating that MIP proteases were unable to cleave IgY bound to GST-Protein-M (Fig. 2B).

### CysP of *M. gallisepticum* was specific for chicken IgY and cleaved IgY bound to both Protein-M and MIB proteins

**Fig 3:**
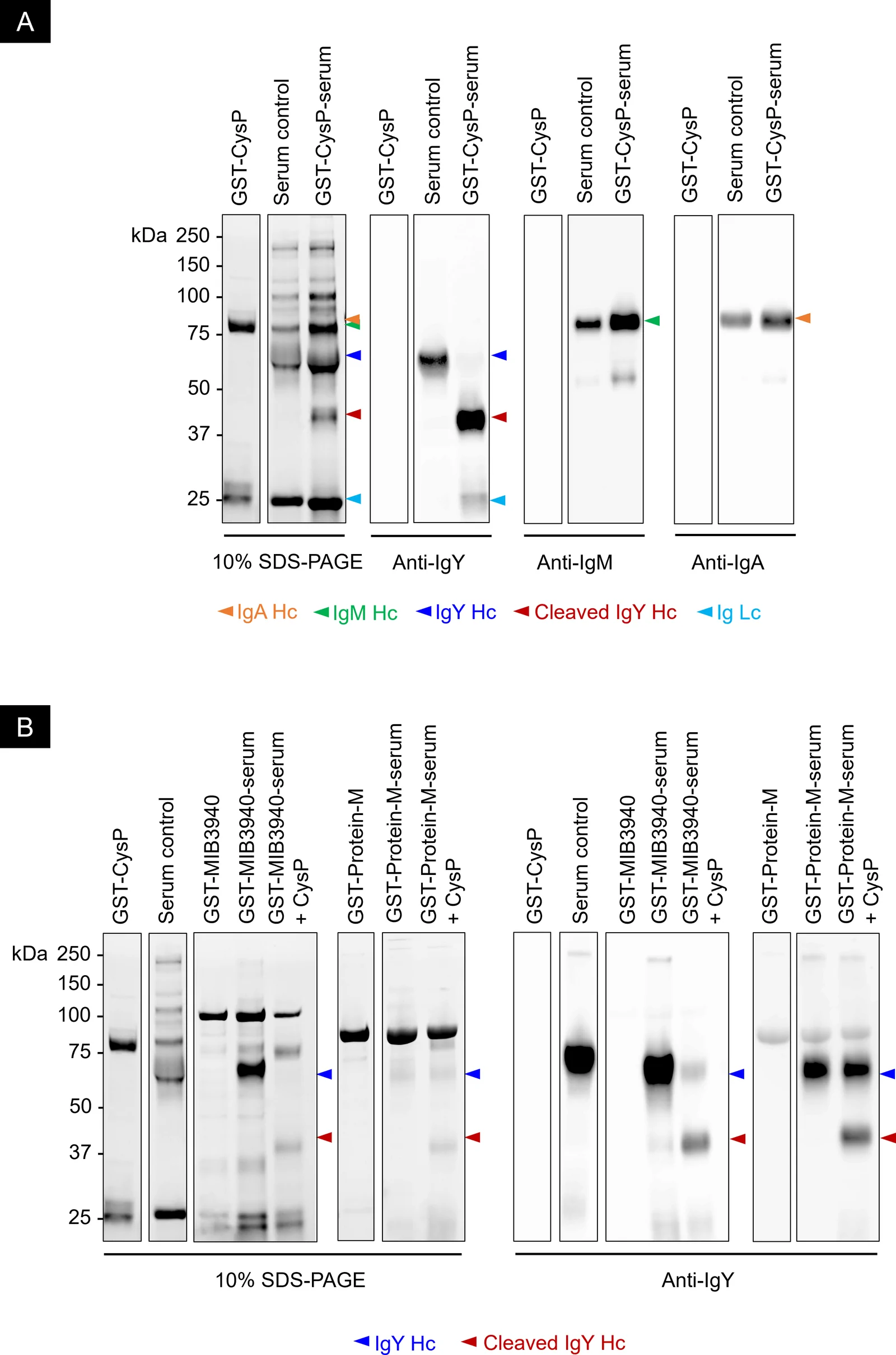
*In vitro* immunoglobulin cleavage activity of CysP. (**A**) CysP cleavage of unbound chicken immunoglobulins. Lanes labeled GST-CysP-serum contain diluted chicken serum that had been incubated with GST-CysP (~80 kDa). The four panels show the 10% polyacrylamide SDS-PAGE gels and Western blots probed with goat antisera against chicken IgY, IgM, or IgA. GST-CysP alone and diluted chicken serum alone were included as controls. Orange arrowheads, ~75 kDa IgA heavy chains; green arrowheads, ~75 kDa IgM heavy chains; dark blue arrowheads, ~65 kDa IgY heavy chains; red arrowheads, ~40–45 kDa cleaved IgY heavy chains; and light blue arrowheads, ~25 kDa IgY light chains. (**B**) CysP cleavage of chicken IgY bound to MIB and Protein-M. The capacity of GST-CysP to cleave chicken IgY complexed with GST-MIB3940 (~100 kDa) or GST-Protein-M (~85 kDa) is shown in the lanes labeled GST-MIB3940-Serum + CysP or GST-Protein-M-Serum + CysP, respectively. The panels on the left show the Coomassie blue-stained 10% polyacrylamide SDS-PAGE gels, and those on the right show Western blots probed with goat anti-chicken IgY. GST-CysP, GST-Protein-M, GST-MIB3940, and diluted chicken serum alone were included as controls. Dark blue arrowheads, ~65 kDa IgY heavy chains; red arrowheads, ~40–45 kDa cleaved IgY heavy chains.

Purified recombinant GST-CysP protein was incubated with diluted chicken serum to assess the proteolytic activity of *M. gallisepticum* CysP. Products were separated by SDS-PAGE, and cleavage of IgY, IgM, and IgA was detected by Western blotting with goat anti-chicken IgY, IgA, or IgM. GST-CysP cleaved the ~65 kDa chicken IgY heavy chain to yield a ~40–45 kDa cleavage product (Fig. 3A). In contrast, the ~75 kDa heavy chains of chicken IgM and IgA remained intact, indicating that GST-CysP did not cleave these immunoglobulins (Fig. 3A). No IgY or IgG cleavage was detected in assays with sera from other avian or mammalian species (Fig. S2B), suggesting that CysP was specific for chicken IgY.

GST-CysP was incubated with eluates from GST-Protein-M and GST-MIB3940 (26) (used as a representative of all MIB proteins in *M. gallisepticum*) binding assays to determine whether CysP could also cleave immunoglobulins bound to *M. gallisepticum* immunoglobulin-binding proteins. SDS-PAGE and Western blot analysis demonstrated that GST-CysP cleaved the ~65 kDa IgY heavy chain when IgY was bound to Protein-M or MIB3940, yielding a ~ 40–45 kDa cleavage product with the Fc domain intact, as was seen in assays with free, unbound IgY (Fig. 3B).

### MIB, MIP, Protein-M, and CysP proteins bound ATP, but only CysP had detectable ATPase activity

**Fig 4:**
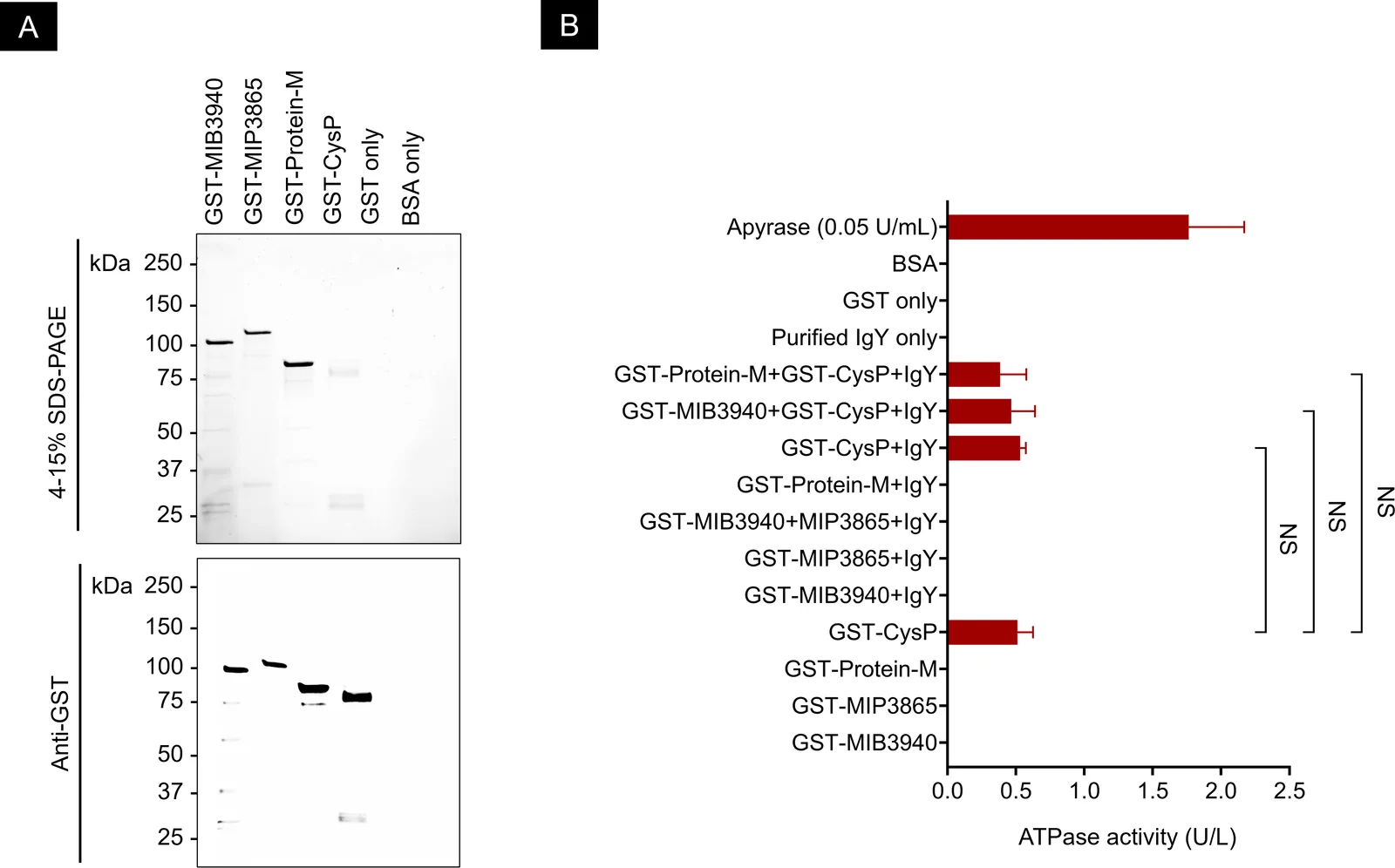
Interaction of Ig-binding and cleavage proteins of *M. gallisepticum* with ATP. (**A**) ATP-binding capacity of MIB-MIP, Protein-M, and CysP proteins. ATP binding by recombinant GST-MIB3940, GST-MIP3865, GST-Protein-M, and GST-CysP was assessed using ATP-agarose pull-down assays. Proteins bound to ATP-agarose beads were analyzed by separating them by SDS-PAGE (top left panel), then blotting them onto PVDF membranes and detecting them with anti-GST antibody (bottom left panel). GST alone and BSA alone were included as negative controls. (**B**) ATPase activity of MIB-MIP, Protein-M, and CysP proteins. ATPase activity of recombinant GST-MIP3865, GST-MIB3940, GST-Protein-M, and GST-CysP was tested alone, as well as in combination with each other and with chicken IgY. Apyrase was included as a positive control, while GST, BSA, and purified IgY alone were included as negative controls. Data represent the mean of three independent experiments with statistical analysis by two-way ANOVA (*p* < 0.05) performed with GraphPad Prism version 9.4.1. NS, no significant difference.

Bioinformatic analysis using ATPint (30) of MIB, MIP, Protein-M, and CysP proteins encoded in the *M. gallisepticum* genome identified ATP-interaction motifs within their amino acid sequences. Based on these findings, ATP-binding capacity was assessed for GST-MIB3940, GST-MIP3865 (recombinant MIB protein of F9C22_03865 of *M. gallisepticum*), GST-Protein-M, and GST-CysP using the ATP-linked agarose method. All four proteins bound to ATP-agarose beads, indicating that they had ATP-binding activity, while no ATP binding was detected with the GST or bovine serum albumin (BSA) negative controls (Fig. 4A).

The capacity of each of these proteins to catalyze ATP hydrolysis was then evaluated using the ATPase/GTPase assay (31). Only GST-CysP had detectable ATPase activity, alone and in combination with unbound purified IgY, as well as with IgY bound to GST-Protein-M or GST-MIB3940 (Fig. 4B). No significant difference in ATPase activity was observed between GST-CysP alone and GST-CysP in combination with purified IgY alone or IgY bound to Ig-binding proteins (*p* > 0.05) (Fig. 4B). ATPase activity was not detected for GST-MIB3940, GST-MIP3865, or GST-Protein-M, whether IgY was or was not present. No activity was detected with the GST, BSA, and purified IgY negative controls (Fig. 4B).

### Reactivity of serum antibodies from chickens infected with MG with MIB-MIP, Protein-M, or CysP proteins

**Fig 5:**
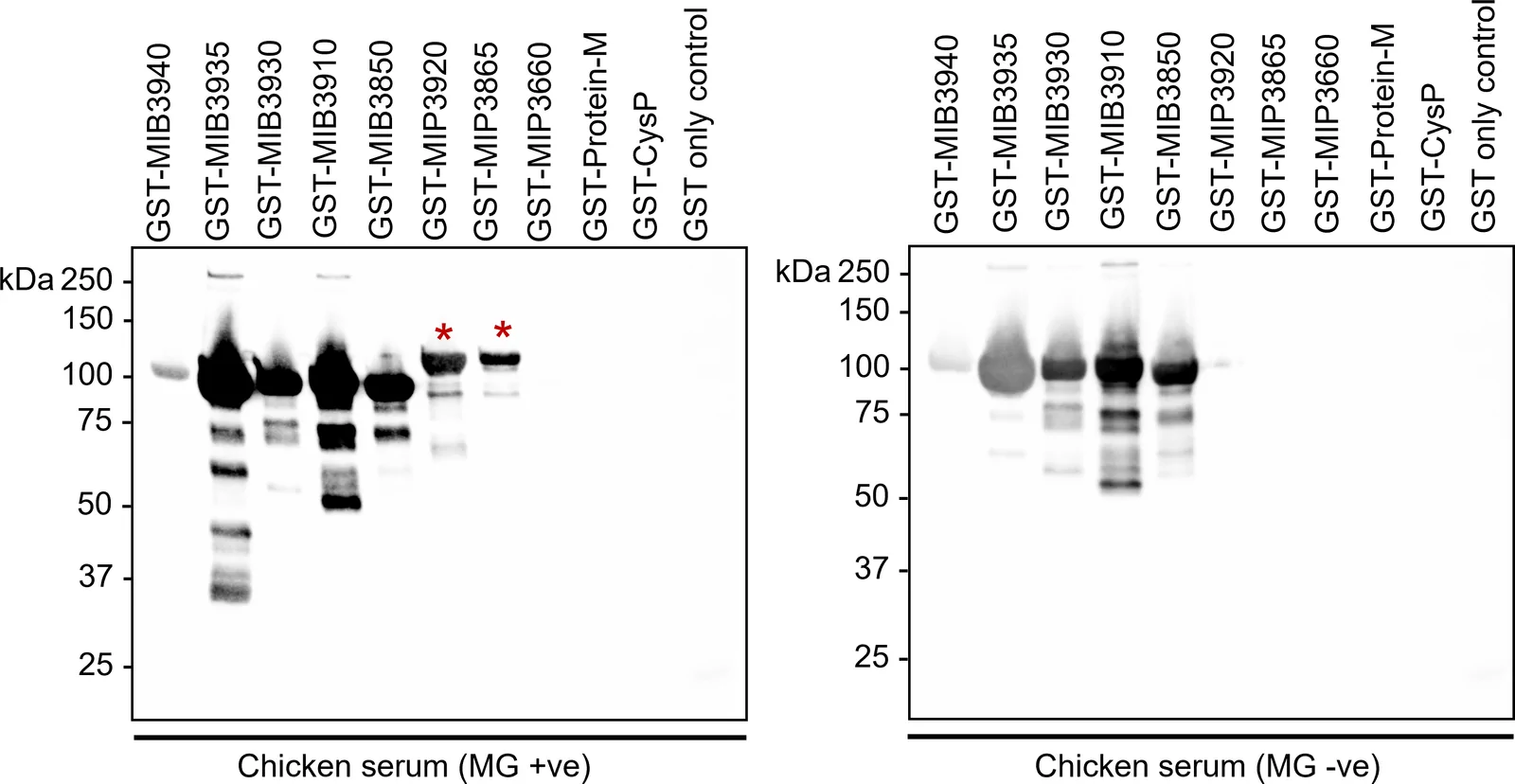
Immunoreactivity of recombinant GST-MIB proteins, GST-MIP proteins, GST-Protein-M, and GST-CysP. Proteins were resolved by 10% polyacrylamide SDS-PAGE, probed with sera from uninfected birds (MG −ve) and birds that had been experimentally infected with *M. gallisepticum* (MG +ve), and chicken IgY that bound to the proteins was detected using goat anti-chicken IgY (H+L) HRP-conjugated antibody. GST alone served as a control. Red asterisks indicate specific immunoreactivity.

The reactivity of antibodies in sera from chickens experimentally infected with *M. gallisepticum* strain Ap3AS with recombinant GST-Protein-M, GST-CysP, GST-MIB, or GST-MIP proteins was assessed by Western blotting. Reactivity was detected with two of the three MIP proteins, GST-MIP3920 (recombinant MIP protein of F9C22_03920 of *M. gallisepticum*), and GST-MIP3865 (26) (Fig. 5). Reactivity was not detected with GST-MIP3660 (recombinant MIP protein encoded by F9C22_03660 of *M. gallisepticum*) (26), GST-Protein-M, or GST-CysP (Fig. 5). Reactivity was observed with all of the GST-MIB proteins, with antibodies in sera from birds infected with MG and from birds that had not been infected, consistent with their immunoglobulin-binding activity (Fig. 5).

### Effect of Ig-binding and Ig-cleavage on Ig-dependent complement-mediated killing of *M. gallisepticum*

A complement killing assay was performed to determine whether *M. gallisepticum* was susceptible to complement-mediated killing in the presence of anti-MG antibodies, and to assess any influence that the presence of Ig-binding or Ig-cleavage proteins might have on this effector mechanism.

Concentrations of viable MG remained stable over the first 6 h in PBS and in heat-inactivated (HI) rabbit anti-*M*. *gallisepticum* serum (RS) (Fig. S3) (Table 1). Dilution of MG in complement alone (C) resulted in a moderate level of killing, with the MG titer decreasing significantly from 7.98 ± 0.12 log_10_ to 5.36 ± 1.19 log_10_ CCU/mL (mean ± standard deviation) within the first 4 h (*p* < 0.05) (Fig. S3) (Table 1). The combination of HI serum and complement (RS + C) resulted in rapid killing, with titers decreasing from 7.88 ± 0.20 to 3.94 ± 0.08 log_10_ CCU/mL within 4 h, significantly lower than was seen with complement alone (*p* < 0.05), confirming that rabbit anti-MG immunoglobulins enhance complement-mediated killing (Table 1) (Fig. S3). Inactivation of complement activity by adding EDTA (RS + C + EDTA) resulted in similar titers to those seen with PBS or RS only. The GST control (RS + C + GST) resulted in similar levels of killing activity to that observed with RS + C, indicating that GST does not interfere with the effect of rabbit IgG on complement-mediated killing (Table 1) (Fig. S3). In contrast, the addition of GST-MIB (RS + C + MIB), GST-MIB + GST-MIP (RS + C + MIB-MIP), or GST-Protein-M (RS + C + Protein-M) significantly reduced the complement-mediated killing compared to that observed for RS + C (*p* < 0.05), resulting in titers of viable MG comparable to those seen for complement alone (Table 1) (Fig. S3). The addition of GST-CysP (RS + C + CysP) alone resulted in titers comparable to those seen for RS + C, but the addition of GST-CysP in combination with GST-Protein-M (RS + C + Protein-M-CysP) or GST-MIB (RS + C + MIB-CysP) resulted in titers similar to those seen for complement alone (Table 1) (Fig. S3).

**TABLE 1:** Comparisons of the effects of Ig-binding and Ig-cleavage proteins on antibody-dependent complement-mediated killing of *M. gallisepticum*^*a*^

Assay components^*b*^	Mean *M. gallisepticum* titer (mean log_10_ CCU/mL ± standard deviation)				
	0 h	2 h	4 h	6 h	12 h
PBS	7.98 ± 0.12^a^	7.98 ± 0.12^a^	7.90 ± 0.08^a^	7.90 ± 0.08^a^	6.90 ± 0.25^a^
RS	8.03 ± 0.06^a^	7.84 ± 0.15^a^	7.82 ± 0.19^a^	7.90 ± 0.08^a^	7.03 ± 0.06^a^
C	7.98 ± 0.12^a^	6.94 ± 0.08^b^	5.36 ± 1.19^b^	5.69 ± 0.07^b^	4.93 ± 0.14^b^
(RS + C)	7.88 ± 0.20^a^	6.09 ± 0.08^c^	3.94 ± 0.08^c^	3.37 ± 0.32^c^	3.2 ± 0.19^c^
(RS + C) + 10 mM EDTA	8.03 ± 0.06^a^	7.82 ± 0.05^a^	7.86 ± 0.24^a^	7.63 ± 0.25^a^	6.80 ± 0.22^a^
(RS + C) + GST	7.94 ± 0.08^a^	6.26 ± 0.28^c^	3.90 ± 0.17^c^	3.44 ± 0.39^c^	3.13 ± 0.11^c^
(RS + C) + MIB	8.03 ± 0.06^a^	7.06 ± 0.06^b^	5.90 ± 0.08^b^	5.22 ± 0.22^b^	4.75 ± 0.22^b^
(RS + C) + MIB-MIP	7.94 ± 0.08^a^	6.90 ± 0.08^b^	5.94 ± 0.13^b^	5.56 ± 0.12^b^	4.71 ± 0.16^b^
(RS + C) + Protein-M	7.91 ± 0.07^a^	6.98 ± 0.12^b^	5.80 ± 0.22^b^	5.34 ± 0.29^b^	4.82 ± 0.19^b^
(RS + C) + CysP	7.94 ± 0.08^a^	6.22 ± 0.22^c^	3.84 ± 0.35^c^	3.36 ± 0.31^c^	3.22 ± 0.22^c^
(RS + C) + MIB-CysP	7.89 ± 0.17^a^	6.81 ± 0.16^b^	5.65 ± 0.19^b^	5.28 ± 0.24^b^	4.59 ± 0.19^b^
(RS + C) + Protein-M-CysP	7.99 ± 0.11^a^	6.58 ± 0.13^bc^	5.76 ± 0.29^b^	5.36 ± 0.24^b^	4.42 ± 0.21^b^

^*a*^ Values marked with the same superscript letter in the same column were not significantly different (*p* > 0.05) in a two-way ordinary ANOVA between groups at each time point.

^*b*^ PBS, phosphate-buffered saline containing 10 mM Ca^2+^ and 0.1 mM Mg^2+^; RS, heat-inactivated rabbit anti-*M*. *gallisepticum* serum; C, guinea pig serum complement; GST, recombinant glutathione S-transferase; MIB, recombinant GST-MIB3930; MIP, recombinant GST-MIP3660; Protein-M, recombinant GST-Protein-M; CysP, recombinant GST-CysP.

## DISCUSSION

These studies characterized the role of two proteins in *M. gallisepticum* that can interact with immunoglobulins: a Protein-M homolog (F9C22_03005) and a cysteine protease, CysP (F9C22_02750), both of which are phylogenetically distinct from the MIB-MIP immunoglobulin evasion system found in the same organism. Protein-M in *M. gallisepticum* retains the leucine-rich repeat structure found in *M. genitalium*, but its amino acid sequence differs markedly from the homologs in the two human pathogens (18, 23). Protein-M homologs are only found in mycoplasma species in the *pneumoniae* phylogenetic cluster, but are highly conserved across *M. gallisepticum* strains and other avian mycoplasmas in the *pneumoniae* cluster, many of which also encode the MIB-MIP system (26). We demonstrated that Protein-M has the capacity to bind IgY, IgM, and IgA from multiple avian hosts of *M. gallisepticum*. Consistent with this broad specificity, Protein-M also bound to mammalian IgG, IgA, and IgM, as has been reported for Protein-M homologs in the human pathogens *M. genitalium* and *M. pneumoniae* (18, 23). *M. gallisepticum* Protein-M bound to avian immunoglobulins in solution, probably via the V_L_ domain, as has been reported for the homologs in human mycoplasmas (12, 23), thereby blocking antigen-antibody binding. The sequence of Protein-M is quite distinct from that of the MIB proteins, and it did not interact with the MIP proteases, which contain mycoplasma-specific DUF31 domains (26), consistent with findings for Protein-M and the DUF31-domain proteases of *M. pneumoniae* (18). This suggests that Protein-M in *M. gallisepticum* functions independently of the MIB-MIP system (26).

Like its *M. synoviae* homolog, the CysP of *M. gallisepticum* cleaved the chicken IgY heavy chain, resulting in separation of the Fab and Fc domains, which would be expected to impair antibody-antigen recognition (17). We demonstrated that CysP in *M. gallisepticum* is specific for chicken IgY *in vitro*, with no detectable activity against IgA or IgM, nor the IgY of other avian species or IgG from mammals, indicating that CysP is unlikely to disrupt immunoglobulins when *M. gallisepticum* infects other avian hosts. Notably, it cleaves both chicken IgY when it is free in solution and when it is bound to Protein-M or the MIB proteins, indicating that it can access and cleave IgY even when it has been captured by Ig-binding proteins. This highlights the potential for functional interactions between these otherwise independent systems, with CysP playing a role in a high-affinity IgY capture-and-cleavage immune evasion mechanism. The close similarity between CysP in *M. gallisepticum* and *M. synoviae*, and the moderate conservation seen across other avian mycoplasmas, is consistent with gene exchange between species sharing avian hosts (17, 32). This conservation, coupled with the absence of homologs in mycoplasmas that are pathogens of mammals and other avian species, suggests that the role of CysP may be specifically in evasion of IgY responses in chickens.

The complement cascade plays a vital role in innate immune defense by mediating bacterial lysis, promoting leukocyte activation, and facilitating phagocytosis via opsonization (33). Antibodies have been shown to enhance complement-mediated killing in bacterial pathogens (34–36), including mycoplasmas, such as the cattle pathogen *Mycoplasma bovis* (37). We showed that the combination of rabbit anti-*M*. *gallisepticum* antibodies and guinea pig complement resulted in substantial and rapid killing of *M. gallisepticum*, whereas complement alone had only a moderate effect on viability. This suggests effective activation of the classical complement pathway in the presence of antibodies, in contrast to the non-specific killing seen with complement alone (33). We also demonstrated that the presence of recombinant MIB protein alone, MIB and MIP in combination, or Protein-M significantly reduced killing by the combination of antibodies and complement, resulting in titers comparable to those seen when *M. gallisepticum* was incubated in complement alone. This demonstrated that these proteins could impair the capacity of rabbit IgG to activate complement, by masking or disrupting antigen-binding sites, thereby preventing antigen-antibody interaction, or by interfering with binding of complement to the Fc region and thus impairing antibody-dependent complement-mediated killing. GST-CysP alone did not impair IgG-dependent complement-mediated killing, consistent with its specificity for chicken IgY. However, when combined with Protein-M or MIB, killing was reduced to the levels seen for complement-only, reflecting the capacity of the Protein-M and MIB proteins to bind IgG (23, 25). It is possible that CysP could impair IgY-dependent complement-mediated killing by removing the complement-activating region when it cleaves the chicken IgY heavy chains to separate the Fab and Fc portions (17). However, this was unable to be determined here, as chicken IgY cannot activate mammalian complement (38, 39). The distribution of MIB-MIP homologs and Protein-M in mycoplasma species infecting mammals (25) suggests that these proteins could interfere with the antibody-dependent complement-mediated killing in mammalian hosts. However, their effects in avian antibody-complement systems require further investigation, as interactions between avian immunoglobulins and serum complement systems are still poorly characterized.

Serum antibodies in chickens experimentally infected with *M. gallisepticum* strain Ap3AS reacted strongly with recombinant MIP3920 and MIP3865, indicating that these MIPs are expressed during infection and are immunogenic, unlike MIP3660, with which no reactivity was detected. This lack of response may reflect variable expression of the MIP proteins during infection (26). Serum antibodies from both infected and uninfected birds bound all five GST-MIB proteins, reflecting their activity as immunoglobulin-binding proteins. Antibodies against recombinant Protein-M were not detected in sera from infected chickens, suggesting that Protein-M may be poorly immunogenic or differentially expressed during infection. Similarly, antibodies against CysP were not detected in sera from infected chickens, although a previous study found that ~75% of chickens infected with *M. synoviae* and >75% infected with *M. gallisepticum* strain S6 had detectable serum antibodies against the CysP of *M. synoviae* (40). It is possible that these differences could reflect phase variation in expression of these proteins, although there are no immediately obvious phase variation mechanisms apparent in the sequences of the genes that encode them in *M. gallisepticum*. Future studies, including targeted transcriptomic and proteomic analyses, in experimentally infected chickens will be useful in understanding whether Protein-M, CysP, and the MIB-MIP proteins are variably expressed *in vivo* and whether this may contribute to long-term immune evasion by *M. gallisepticum*.

The relationship between immunoglobulin binding and cleavage proteins and ATPases encoded in close genomic proximity to the MIB-MIP system genes has been investigated recently (26, 41, 42), and these studies have suggested a role for ATP in antibody binding and cleavage. The studies reported here demonstrated that the MIB-MIP proteins, Protein-M, and the CysP of *M. gallisepticum* can all bind ATP. The ability of the MIB-MIP proteins to bind, but not hydrolyze, ATP may reflect a dependence on closely associated ATPases (26), which may catalyze ATP hydrolysis and facilitate the functions of the MIB-MIP system. In contrast, CysP had ATPase activity *in vitro*, alone and in the presence of Ig-binding proteins or IgY. While the role of ATP hydrolysis in these systems is yet to be fully established, it is notable that CysP can catalyze it independently of any other proteins.

In summary, this study has expanded our understanding of immune evasion in *M. gallisepticum* by identifying Protein-M and CysP as two additional proteins that can target immunoglobulins and can function independently of the MIB-MIP system (26). Protein-M exhibits broad immunoglobulin-binding activity across both avian and mammalian species, whereas CysP only cleaves chicken IgY, suggesting it is adapted to specifically interfere with the immune response of chickens. While these three systems can function independently *in vitro*, their potential synergistic or competitive interactions, and their expression in the host during infection, remain to be explored. Both Protein-M and the MIB-MIP proteins impaired antibody-dependent complement-mediated killing *in vitro*, highlighting their capacity to disrupt a key effector mechanism of humoral immunity (11). The capacity of the proteins in all three systems to bind ATP, and the intrinsic ATPase activity of CysP, suggest the involvement of ATP hydrolysis in the functions of these systems *in vivo*. Collectively, these findings highlight the complexity of the interactions between *M. gallisepticum* and its host, and the need to further examine the interplay between the multiple, distinct immune evasion strategies employed by mycoplasmas, and underscore the evolutionary advantage of maintaining complementary mechanisms that enable their survival and persistence in the host under sustained host immune pressure.

## MATERIALS AND METHODS

### Structural, functional, and homology prediction

Putative Protein-M and CysP homologs in *M. gallisepticum* were identified using BLASTX and the sequences of the *M. genitalium* Ig-blocking Protein-M (MG_281; GenBank protein accession, WP_338427464.1) and the *M. synoviae* cysteine protease (CysP; GenBank protein accession, ACL13547.1) as query sequences using the default settings (17, 23). The nucleotide and protein sequences of the putative Protein-M and CysP homologs in *M. gallisepticum* strain Ap3AS (GenBank CP044624.1) were identified and retrieved using Genious Prime 2023.0.1.

The sequences of the Protein-M and CysP homologs were characterized using DeepTMHMM analysis for cellular topology prediction (29), Conserved Domain Search (43, 44) to identify predicted functional domains, and Phyre2 analysis (45) to predict putative functions and structures. HHpred analysis in the MPI Bioinformatics toolkit was used to detect remote homology using the PDB database, as updated on 14th March 2026, using the default settings (27, 46). ATPint was used for sequence-based identification of predicted ATP-binding residues (30). The ProtParam (Expasy) tool was used to determine the predicted amino acid length and molecular mass (kDa) of the proteins (47).

To identify homologs of Protein-M and CysP in other avian mycoplasma species and additional strains of *M. gallisepticum*, BLASTX searches were performed under default settings using the Mold, Protozoan, and Coelenterate Mitochondrial and Mycoplasma/Spiroplasma genetic code. The nucleotide sequences of the Protein-M and CysP gene homologs from *M. gallisepticum* Ap3AS were used as queries, and putative protein homologs were retrieved from the NCBI database on 16 March 2026 (48).

### Bacterial strains and media

High-efficiency NEB 5-alpha competent *Escherichia coli* cells (NEB, USA) were used for plasmid maintenance, and BL21 (DE3) competent *E. coli* cells (NEB) were used for expression of the recombinant proteins. Plasmid-transformed *E. coli* cells were cultured at 37°C in Luria-Bertani (LB) broth containing 1% wt/vol tryptone (Oxoid, UK), 0.5% wt/vol yeast extract (Oxoid), and 0.5% wt/vol NaCl (Ajax Finechem, Australia) for 18 h with shaking at 200 rpm or on LB agar containing 1% bacteriological agar (Oxoid) in LB broth and ampicillin at 50 µg/mL (Sigma-Aldrich, USA). *E. coli* cultures in LB broth were stored in 40% glycerol (Ajax FineChem) at −80°C. *E. coli* cell pellets were frozen at −20°C for short-term storage and at −80°C for long-term storage.

*M. gallisepticum* strain Ap3AS was obtained from the Asia-Pacific Centre for Animal Health (APCAH) culture collection at The University of Melbourne, Victoria, Australia. The Ap3AS stock culture was diluted 1:20 in *M. gallisepticum* (MG) medium containing 0.75% wt/vol tryptone peptone (Difco, USA), 0.25% wt/vol phytone peptone (Gibco, USA), 0.05% wt/vol proteose peptone (Bacto, USA), 0.5% wt/vol yeast extract (Difco), 0.5% wt/vol NaCl (Chem-supply, AU), 0.04% wt/vol KCl (Ajax Fine-Chem), 0.035% wt/vol MgSO_4_·7H_2_O (Ajax Fine-Chem), 0.005% wt/vol Na_2_HPO_4_ (Chem-supply), 0.01% KH_2_PO_4_ (Chem-supply), 0.1% wt/vol glucose (Millipore, USA), 0.2% herring sperm DNA (Invitrogen, USA), 1% yeast autohydrolysate (freshly prepared in-house from commercially manufactured baker’s yeast [Fermex Distributors, Victoria, Australia]), 10% vol/vol heat-inactivated swine serum (Selborne Biological Services, Australia), 0.0048% phenol red solution (Chroma-Gesellschaft, Germany), and penicillin G (Sigma Aldrich, USA) at 0.3 mg/mL, with the pH adjusted to 8.1. Cultures were incubated at 37°C for 18 h to reach the stationary phase.

### DNA cloning, recombinant protein expression, and purification

Cloning, protein expression, and purification of recombinant proteins were performed as described previously (26). Briefly, coding sequences from *M. gallisepticum* Ap3AS were modified for expression in *E. coli* by replacing TGA codons with TGG, removing the SignalP-6.0 predicted signal peptide regions, and introducing BamHI (5′ end) and SalI (3′ end) restriction endonuclease cleavage sites to facilitate cloning into pGEX-4T-1 (Cytiva, USA). Synthetic gene constructs in pUC57 plasmids (GenScript, USA) were digested, gel-purified, and ligated into the pGEX-4T-1 expression vector, which was then used to transform competent NEB 5-alpha *E. coli* cells, with transformants selected on ampicillin LB agar. Ampicillin-resistant colonies were screened for inserts of the expected size by plasmid extraction, restriction endonuclease digestion, and 1% agarose gel electrophoresis. The identity of the inserts was confirmed by Sanger sequencing using pGEX_F (forward 5′-CCAGCAAGTATATAGCATGGCC-3′) and pGEX_R (reverse 5′-CTCCGGGAGCTGCATGTG-3′) primers. Sequence-confirmed plasmids were used to transform competent BL21 (DE3) *E. coli* cells, and the plasmids in the transformants were validated by plasmid extraction and restriction endonuclease digestion prior to protein expression. Overnight cultures of transformed BL21 (DE3) *E. coli* cells in LB broth were diluted 1:100, incubated to mid-log phase and induced with 1 mM IPTG (Sigma-Aldrich) to express the GST-tagged recombinant proteins. The *E. coli* cells were harvested, lysed with lysozyme (1 mg/mL) and sonication (25–30 short bursts of 15 s each, on ice), treated with 0.2% Triton X-100, and soluble proteins were purified using glutathione Sepharose columns (Cytiva). GST-tagged recombinant proteins were eluted from the column using glutathione elution buffer, dialyzed against 1× PBS at 4°C, and stored at −80°C. The GST-MIB and GST-MIP proteins and GST alone were also expressed and purified as described previously (26). Protein concentrations were determined using the Pierce BCA Protein Assay (Thermo Fisher Scientific, USA), and expected molecular masses were calculated using ProtParam (Expasy), with 26 kDa added to account for the molecular mass of the GST tag.

### SDS-PAGE analysis and Western blotting

SDS-PAGE analysis and Western blotting were performed as described previously (26). Briefly, protein samples in an equal volume of 2× reducing lysis buffer were resolved by 10% polyacrylamide SDS-PAGE alongside Precision Plus Protein Western-C Standards (Bio-Rad, USA), and the proteins were then transferred to a PVDF membrane (Bio-Rad). Membranes were blocked with 5% (wt/vol) skim milk powder in PBST (1× PBS, 0.1% [vol/vol] Tween-20 [Chem-supply]), washed with PBST and probed as previously described (26). GST-fusion proteins were detected using goat anti-GST antibody (GE Healthcare, UK) and HRP-conjugated polyclonal rabbit anti-goat antibodies (Dako, USA). Chicken IgY was detected using HRP-conjugated goat anti-chicken IgY (H+L) (Bio-Rad; 1:2,000) or IgY Fc (Bio-Rad; 1:2,000) antibodies. Chicken IgM was detected with goat anti-chicken IgM antibodies (Bio-Rad; 1:1,500) and HRP-conjugated polyclonal rabbit anti-goat secondary antibodies (Dako; 1:2,000). Chicken IgA was detected with HRP-conjugated goat anti-chicken IgA antibodies (Bio-Rad; 1:600). Blots were developed using Clarity Western ECL Substrate (Bio-Rad) and imaged using the ChemiDoc MP system (Bio-Rad).

### Animal sera

The animal sera used in this study were obtained from various members of the Asia-Pacific Centre for Animal Health (APCAH) at The University of Melbourne (chicken serum from Dr. Pollob Shil, turkey, pheasant and pig sera from Anna Kanci Condello, goat serum from Assoc. Prof. Simon Firestone, sheep serum from Dr. Nadeeka Wawegama, rat and rabbit sera from Assoc. Prof. Carol Hartley, horse serum from Kim Carey, and tammar wallaby serum from Dr. Paola Vaz). The duck serum was obtained from Prof. Marcel Klaassen from the School of Life and Environmental Sciences, Deakin University.

Sera from chickens experimentally infected with *M. gallisepticum* strain Ap3AS and from uninfected chickens that were used in this study were obtained from an unrelated study conducted at The University of Melbourne (unpublished data) under the approval of The University of Melbourne Animal Ethics Committee (Ethics approval number: 20256) and conducted in accordance with relevant institutional and national guidelines for the care and use of animals. Specific-pathogen-free (SPF) White Leghorn chicks were hatched from fertile eggs supplied by Australian SPF Services Pty. Ltd. (Woodend, Victoria, Australia) and raised at the Asia-Pacific Centre for Animal Health (APCAH) animal trial facility at The University of Melbourne Veterinary Clinical Centre (Werribee, Victoria, Australia). The chicks were randomly allocated into two groups: Group 1, negative control (unvaccinated and unchallenged) (*n* = 10); and Group 2, challenged only (*n* = 10). Each group was housed in a separate HEPA-filtered fiberglass isolator unit under negative pressure, with feed and water provided *ad libitum*. All birds were wing-tagged with a unique number as a means of identification. At 8 weeks of age, the birds in Group 2 were challenged with the virulent Ap3AS strain by nebulization of approximately 40 mL of a culture containing 10^8^ CCU/mL using compressed air into a purpose-built infection chamber (49). Birds were monitored for clinical signs of respiratory disease associated with infection with *M. gallisepticum* over the duration of the experiment. Two weeks after infection, all the birds were euthanized by intravenous barbiturate overdose, and blood samples were collected for serum preparation.

No additional animal experiments were performed as part of this study. Polyclonal rabbit antiserum against a *M. gallisepticum* whole-cell lysate was obtained from existing collections in the Mycoplasma Laboratory, Asia-Pacific Centre for Animal Health, Melbourne Veterinary School, University of Melbourne.

### Immunoglobulin-binding assays

The immunoglobulin-binding assay was performed as previously described (26). Briefly, recombinant GST-Protein-M (0.5 mM final concentration) and GST-MIB3940 (F9C22_03940) (used as a representative of all MIB proteins in *M. gallisepticum*) were incubated overnight with filtered chicken serum diluted 1:10 in 1× PBS. Bound complexes were purified using a GST SpinTrap column (Cytiva, USA). Protein samples eluted from the column were analyzed by 10% polyacrylamide SDS-PAGE, followed by Western blotting, as described above. Control reactions included diluted serum without a recombinant protein and GST-Protein-M or GST-MIB3940 alone. The assay was also performed with purified chicken IgY (Bio-Rad) and pooled sera from turkeys, pheasants, ducks, goats, sheep, rabbits, horses, pigs, and tammar wallabies to assess cross-species binding.

### Immunoglobulin cleavage assays

The immunoglobulin cleavage activity of CysP was assessed by incubating filtered chicken serum diluted 1:10 in 1× PBS with recombinant GST-CysP (0.5 mM final concentration) at 37°C for 4 h. The protein products were resolved by 10% polyacrylamide SDS-PAGE, and cleaved IgY, IgM, or IgA was detected by probing Western blots using the immunoglobulin-specific antibodies described above. Cross-species activity was evaluated by performing the assay using pooled sera from turkeys, pheasants, ducks, goats, sheep, rabbits, horses, pigs, and tammar wallabies.

To evaluate the proteolytic activity of recombinant GST-CysP on immunoglobulins bound to GST-Protein-M or GST-MIB3940, eluates from the binding assays were incubated separately with 0.5 mM GST-CysP at 37°C for 4 h, the proteins were separated by 10% polyacrylamide SDS-PAGE, transferred onto PVDF membranes, and then probed using HRP-conjugated anti-chicken IgY Fc antibodies as described above.

The proteolytic activity of the MIP proteins MIP3920 (F9C22_03920), MIP3865 (F9C22_03865), and MIP3660 (F9C22_03660) on purified Protein-M-bound IgY complexes was assessed by incubating each GST-tagged MIP (1 mM) with Protein-M-IgY eluates at 37°C for 6 h, followed by 10% polyacrylamide SDS-PAGE and Western blotting as described above. All assays included appropriate negative controls, including recombinant GST-proteins and diluted chicken serum alone.

### ATP-binding assay

The ATP-binding capacity of recombinant GST-tagged MIP, MIB, CysP, and Protein-M proteins was assessed using an ATP-agarose pull-down method described previously (31). Briefly, GST-MIP3865, GST-MIB3940, GST-Protein-M, and GST-CysP were each mixed with 150 µL of 4% ATP-conjugated agarose beads (Sigma-Aldrich), and the mixture was diluted to a final volume of 300 µL in binding buffer (50 mM Tris-HCl, pH 7.5, and 10 mM MgCl_2_). Samples were incubated at 4°C for 2 h with gentle mixing every 30 min. Beads were then washed five times with cold TBS-T (20 mM Tris pH 7.4, 150 mM NaCl, and 0.1% vol/vol Tween-20) and pelleted by centrifugation at 12,000 × *g* for 5 min. Bound proteins were eluted in 2× reducing lysis buffer by incubation at 100°C for 5 min and then separated by 10% polyacrylamide SDS-PAGE. Proteins were transferred onto PVDF membranes, and the GST tag on the recombinant proteins was detected as described above. GST alone and BSA were included as negative controls.

### ATPase assay

The recombinant proteins were assayed for ATPase activity as described previously (31), with minor modifications. Briefly, purified recombinant proteins (GST-MIP3865, GST-MIB3940, GST-Protein-M, and GST-CysP) were dialyzed against 1× TBS to remove residual phosphate ions. The assay was performed using an ATPase/GTPase Activity Assay Kit (Sigma-Aldrich) according to the manufacturer’s instructions. Each protein (0.5 mM final concentration) was tested alone or in combination with 0.5 mM purified chicken IgY diluted in distilled water, with volumes standardized using the assay buffer. Apyrase (0.05 U/mL; NEB) served as a positive control, while GST, BSA, and IgY alone were included as negative controls. Prior to the assay, each reaction mixture (40 µL) was tested for free phosphate by adding 200 µL of Malachite Green reagent, and only samples with an absorbance <0.3 at 620 nm, indicating low concentrations of free phosphate, were included. To measure ATPase activity, reactions were incubated with 1 mM ATP (Sigma-Aldrich) for 4 h at room temperature in a 96-well microtiter plate. Malachite Green reagent was added, and samples were incubated for another hour before measuring the absorbance at 620 nm using a FLUOstar Omega plate reader (BMG Labtech, Germany). Background absorbance from blank reactions (with no test protein) was subtracted from the test readings. All reactions were performed in triplicate, and ATPase activity was calculated using a phosphate standard curve. Three independent experiments were conducted. Statistical comparisons were made using unpaired two-way ANOVAs (GraphPad Prism, v9.4.1), with *p* < 0.05 considered significant.

### Screening immunoreactivity of proteins

To assess immunoreactivity, recombinant GST-Protein-M, GST-CysP, five GST-MIB proteins, and three functional GST-MIP proteins (26) were resolved by 10% polyacrylamide SDS-PAGE and transferred to PVDF membranes. Recombinant GST alone served as the negative control. Blots were probed with pooled chicken sera from birds experimentally infected with *M. gallisepticum* strain Ap3AS (diluted 1:1,000, for 1 h), followed by incubation with HRP-conjugated goat anti-chicken IgY (H+L) antibodies (Bio-Rad; 1:2,000, 1 h). Blots were developed as described above. Pooled sera from uninfected birds were used as the negative control.

### Complement-mediated killing inhibition assay

To evaluate the ability of MIB, MIP, CysP, and Protein-M to interfere with antibody-dependent complement-mediated killing of *M. gallisepticum*, a series of *in vitro* complement-mediated killing assays were performed with recombinant GST-fusion proteins. Polyclonal rabbit antiserum raised against a whole-cell lysate of *M. gallisepticum* strain Ap3AS was used as the source of immunoglobulins. The presence of anti-*M*. *gallisepticum* antibodies was confirmed by SDS-PAGE and Western blotting. Briefly, whole *M. gallisepticum* strain Ap3AS cells (4 × 10^8^ CCU/mL) were lysed by incubation in 5 µL of 2× reducing lysis buffer for 5 min at 100°C, and the proteins were separated by SDS-PAGE in a 4–15% polyacrylamide gradient gel (Bio-Rad). Western blotting was performed as described above, and the blots were incubated in rabbit anti-*M*. *gallisepticum* antisera (diluted 1:100, for 1 h), followed by HRP-conjugated swine anti-rabbit Ig antibodies (Dako; diluted 1:2,000, for 1 h). Upon confirming immunoreactivity, the serum was diluted 1:10 in sterile 1× PBS, passed through a 0.45-µm Millex-HV PVDF low protein-binding filter (Sigma-Aldrich), and heat-inactivated at 56°C for 30 min. Guinea pig serum complement (Sigma-Aldrich) was diluted 1:30 in 1× PBS, filtered as described above, and used immediately.

Ig-binding was assessed using GST-Protein-M and GST-MIB3930, while Ig-cleavage was tested with GST-MIB3930 + GST-MIP3660 (the only MIP in *M. gallisepticum* able to cleave rabbit IgG), GST-CysP, GST-Protein-M + GST CysP, and GST-MIB3930 + GST CysP. All proteins were used at a final concentration of 10 µM. Binding and cleavage were confirmed by SDS-PAGE and Western blotting using the specific antibodies described above. GST alone was used as the negative control.

The complement-mediated killing assay was performed using a method described previously (37), with minor modifications. Briefly, cells in 100 µL of a culture of *M. gallisepticum* strain Ap3AS (~4 × 10^8^ CCU/mL) were resuspended in 100 µL of 1× PBS containing 10 mM Ca^2+^ and 0.1 mM Mg^2+^, and the mixture was incubated with 100 µL of heat-inactivated antiserum, either alone or in combination with the recombinant proteins, at 37°C for 30 min. Reaction volumes were standardized to 300 µL using sterile PBS before 100 µL of the diluted guinea pig complement was added. Samples were collected at defined time points (0, 2, 4, 6, and 12 h) after addition of the complement. Controls included *M. gallisepticum* in PBS alone, in complement alone, in antiserum alone, and in antiserum plus complement and 10 mM EDTA to inhibit complement activity. Viable mycoplasma titers were determined at each time point and expressed as color-changing units (CCU)/mL using previously established methods (50). All experiments were performed with three biological replicates, titers were log_10_ transformed, and statistical comparisons were made using ordinary two-way ANOVAs (with Tukey’s multiple comparison tests) (GraphPad Prism v9.4.1), with *p* < 0.05 considered significant.

## SUPPLEMENTAL MATERIAL

Supplemental figures iai.00373-26-s0001.docx Fig. S1 to S3.

Supplemental tables iai.00373-26-s0002.docx Tables S1 to S4.

ASM does not own the copyrights to Supplemental Material that may be linked to, or accessed through, an article. The authors have granted ASM a non-exclusive, world-wide license to publish the Supplemental Material files. Please contact the corresponding author directly for reuse.

## References

[1] Razin S. 1978. The mycoplasmas. Microbiol Rev 42:414–470. doi:10.1128/mr.42.2.414-470.1978 PMC281436 353482

[2] Baseman JB, Tully JG. 1997. Mycoplasmas: sophisticated, reemerging, and burdened by their notoriety. Emerg Infect Dis 3:21–32. doi:10.3201/eid0301.970103 PMC2627593 9126441

[3] Razin S, Yogev D, Naot Y. 1998. Molecular biology and pathogenicity of mycoplasmas. Microbiol Mol Biol Rev 62:1094–1156. doi:10.1128/MMBR.62.4.1094-1156.1998 PMC98941 9841667

[4] Pitcher DG, Nicholas RAJ. 2005. *Mycoplasma* host specificity: fact or fiction? Vet J 170:300–306. doi:10.1016/j.tvjl.2004.08.011 16266844

[5] Ley DH, Ley HW. 1997. Mycoplasma gallisepticum infection, p 807–833. *In* Saif YM, Fadly AM, Glisson JR, McDougald LR, Nolan LK, Swayne DE (ed), Diseases of Poultry, 12 ed. Blackwell.

[6] Stipkovits L, Kempf I. 1996. Mycoplasmoses in poultry. Rev Sci Tech 15:1495–1525. doi:10.20506/rst.15.4.986 9190023

[7] Chhabra PC, Goel MC. 1981. Immunological response of chickens to *Mycoplasma gallisepticum* infection. Avian Dis 25:279–293. doi:10.2307/1589922 7259674

[8] Simecka JW, Ross SE, Cassell GH, Davis JK. 1993. Interactions of mycoplasmas with B cells: antibody production and nonspecific effects. Clin Infect Dis 17:S176–S182. doi:10.1093/clinids/17.supplement_1.s176 8399911

[9] Fernald GW, Clyde WA, Denny FW. 1967. Nature of the immune response to *Mycoplasma pneumoniae*. J Immunol 98:1028–1038. doi:10.4049/jimmunol.98.5.1028 4164844

[10] Javed MA, Frasca S Jr, Rood D, Cecchini K, Gladd M, Geary SJ, Silbart LK. 2005. Correlates of immune protection in chickens vaccinated with *Mycoplasma gallisepticum* strain GT5 following challenge with pathogenic *M. gallisepticum* strain R(low). Infect Immun 73:5410–5419. doi:10.1128/IAI.73.9.5410-5419.2005 PMC1231071 16113257

[11] Arfi Y, Lartigue C, Sirand-Pugnet P, Blanchard A. 2021. Beware of *Mycoplasma* anti-immunoglobulin strategies. mBio 12:e0197421. doi:10.1128/mBio.01974-21 PMC8593674 34781733

[12] Qin L, Chen Y, You X. 2019. Subversion of the immune response by human pathogenic *Mycoplasmas*. Front Microbiol 10:1934. doi:10.3389/fmicb.2019.01934 PMC6712165 31497004

[13] Kilian M, Mestecky J, Russell M. 1988. Defense mechanisms involving Fc-dependent functions of immunoglobulin a and their subversion by bacterial immunoglobulin A proteases. Microbiol Rev 52:296–303. doi:10.1128/mr.52.2.304-304.1988 PMC373140 3045518

[14] Potempa J, Pike RN. 2009. Corruption of innate immunity by bacterial proteases. J Innate Immun 1:70–87. doi:10.1159/000181144 PMC2743019 19756242

[15] Kilian M, Brown MB, Brown TA, Freundt EA, Cassell GH. 1984. Immunoglobulin A1 protease activity in strains of *Ureaplasma urealyticum*. Acta Pathol Microbiol Scand B Microbiol 92:61–64. doi:10.1111/j.1699-0463.1984.tb02794.x 6369873

[16] Robertson JA, Stemler ME, Stemke GW. 1984. Immunoglobulin a protease activity of *Ureaplasma urealyticum*. J Clin Microbiol 19:255–258. doi:10.1128/jcm.19.2.255-258.1984 PMC271032 6365962

[17] Cizelj I, Berčič RL, Dušanić D, Narat M, Kos J, Dovč P, Benčina D. 2011. *Mycoplasma gallisepticum* and *Mycoplasma synoviae* express a cysteine protease CysP, which can cleave chicken IgG into Fab and Fc. Microbiology (Reading) 157:362–372. doi:10.1099/mic.0.045641-0 20966088

[18] Blötz C, Singh N, Dumke R, Stülke J. 2020. Characterization of an immunoglobulin binding protein (IbpM) from *Mycoplasma pneumoniae*. Front Microbiol 11:685. doi:10.3389/fmicb.2020.00685 PMC7176901 32373096

[19] Boyle MD. 1990. *Bacterial Immunoglobulin-Binding Proteins*, p 1–21. *In* Bacterial Immunoglobulin-binding Proteins. Elsevier.

[20] Fischetti VA. 1989. Streptococcal M protein: molecular design and biological behavior. Clin Microbiol Rev 2:285–314. doi:10.1128/CMR.2.3.285 PMC358122 2670192

[21] Sjöbring U, Björck L, Kastern W. 1991. Streptococcal protein G. gene structure and protein binding properties. J Biol Chem 266:399–405. doi:10.1016/S0021-9258(18)52448-0 1985908

[22] Graille M, Stura EA, Corper AL, Sutton BJ, Taussig MJ, Charbonnier J-B, Silverman GJ. 2000. Crystal structure of a *Staphylococcus aureus* protein a domain complexed with the fab fragment of a human IgM antibody: structural basis for recognition of B-cell receptors and superantigen activity. Proc Natl Acad Sci USA 97:5399–5404. doi:10.1073/pnas.97.10.5399 PMC25840 10805799

[23] Grover RK, Zhu X, Nieusma T, Jones T, Boreo I, MacLeod AS, Mark A, Niessen S, Kim HJ, Kong L, Assad-Garcia N, Kwon K, Chesi M, Smider VV, Salomon DR, Jelinek DF, Kyle RA, Pyles RB, Glass JI, Ward AB, Wilson IA, Lerner RA. 2014. A structurally distinct human mycoplasma protein that generically blocks antigen-antibody union. Science 343:656–661. doi:10.1126/science.1246135 PMC3987992 24503852

[24] Moussa A, Nir-Paz R, Rottem S. 2009. Binding of IgA by *Mycoplasma penetrans*. Curr Microbiol 58:360–365. doi:10.1007/s00284-009-9359-1 19189179

[25] Arfi Y, Minder L, Di Primo C, Le Roy A, Ebel C, Coquet L, Claverol S, Vashee S, Jores J, Blanchard A, Sirand-Pugnet P. 2016. MIB-MIP is a mycoplasma system that captures and cleaves immunoglobulin G. Proc Natl Acad Sci USA 113:5406–5411. doi:10.1073/pnas.1600546113 PMC4868467 27114507

[26] Ekanayake D, Vaz P, Legione AR, Wawegama NK, Browning GF, Tivendale KA. 2026. Host specificity and horizontal gene transfer in the MIB-MIP immunoglobulin evasion system in *Mycoplasma gallisepticum**.* Vet Res 57:145. doi:10.1186/s13567-026-01779-x PMC13459921 42581367

[27] Zimmermann L, Stephens A, Nam S-Z, Rau D, Kübler J, Lozajic M, Gabler F, Söding J, Lupas AN, Alva V. 2018. A Completely reimplemented MPI bioinformatics toolkit with a new HHpred server at its core. J Mol Biol 430:2237–2243. doi:10.1016/j.jmb.2017.12.007 29258817

[28] Söding J, Biegert A, Lupas AN. 2005. The HHpred interactive server for protein homology detection and structure prediction. Nucleic Acids Res 33:W244–W248. doi:10.1093/nar/gki408 PMC1160169 15980461

[29] Hallgren J, Tsirigos KD, Pedersen MD, Almagro Armenteros JJ, Marcatili P, Nielsen H, Krogh A, Winther O. 2022. DeepTMHMM predicts alpha and beta transmembrane proteins using deep neural networks. bioRxiv. doi:10.1101/2022.04.08.487609

[30] Chauhan JS, Mishra NK, Raghava GPS. 2009. Identification of ATP binding residues of a protein from its primary sequence. BMC Bioinformatics 10:434. doi:10.1186/1471-2105-10-434 PMC2803200 20021687

[31] Adamu JY, Mitiku F, Hartley CA, Sansom FM, Marenda MS, Markham PF, Browning GF, Tivendale KA. 2020. *Mycoplasma bovis mbfN* encodes a novel LRR lipoprotein that undergoes proteolytic processing and binds host extracellular matrix components. J Bacteriol 203:10–1128. doi:10.1128/JB.00154-20 PMC7950404 33077633

[32] Noormohammadi AH, Markham PF, Duffy MF, Whithear KG, Browning GF. 1998. Multigene families encoding the major hemagglutinins in phylogenetically distinct mycoplasmas. Infect Immun 66:3470–3475. doi:10.1128/IAI.66.7.3470-3475.1998 PMC108374 9632627

[33] Merle NS, Church SE, Fremeaux-Bacchi V, Roumenina LT. 2015. Complement system part i - molecular mechanisms of activation and regulation. Front Immunol 6:262. doi:10.3389/fimmu.2015.00262 PMC4451739 26082779

[34] Muts RM, den Boer MA, Bardoel BW, Aerts PC, de Haas CJC, Heck AJR, Rooijakkers SHM, Heesterbeek DAC. 2023. Artificial surface labelling of *Escherichia coli* with streptagii antigen to study how monoclonal antibodies drive complement-mediated killing. Sci Rep 13:18836. doi:10.1038/s41598-023-46026-x PMC10620216 37914798

[35] Thiriard A, Raze D, Locht C. 2018. Diversion of complement-mediated killing by *Bordetella**.* Microbes Infect 20:512–520. doi:10.1016/j.micinf.2018.02.002 29454132

[36] Drogari-Apiranthitou M, Kuijper EJ, Dekker N, Dankert J. 2002. Complement activation and formation of the membrane attack complex on serogroup B Neisseria meningitidis in the presence or absence of serum bactericidal activity. Infect Immun 70:3752–3758. doi:10.1128/IAI.70.7.3752-3758.2002 PMC128098 12065518

[37] Zhang Y-K, Li X, Zhao H-R, Jiang F, Wang Z-H, Wu W-X. 2019. Antibodies specific to membrane proteins are effective in complement-mediated killing of *Mycoplasma bovis*. Infect Immun 87:10–1128. doi:10.1128/IAI.00740-19 PMC6867846 31548318

[38] Carlander D, Larsson A. 2001. Avian antibodies can eliminate interference due to complement activation in ELISA. Ups J Med Sci 106:189–195. doi:10.3109/2000-1967-145 12166511

[39] Marquardt WW. 1973. Avian complement-fixation. Avian Dis 17:236–250. 4572572

[40] Cizelj I, Berčič RL, Dušanić D, Benčina M, Narat M, Zorman-rojs O, Benčina D. 2013. Poultry infected with *Mycoplasma gallisepticum* or *Mycoplasma synoviae* produce antibodies to their cysteine protease CysP. Acta Agric Slov 102:19–27. doi:10.14720/aas.2013.102.1.14903

[41] Nottelet P, Bataille L, Gourgues G, Anger R, Lartigue C, Sirand-Pugnet P, Marza E, Fronzes R, Arfi Y. 2021. The *Mycoplasma* surface proteins MIB and MIP promote the dissociation of the antibody-antigen interaction. Sci Adv 7:eabf2403. doi:10.1126/sciadv.abf2403 PMC7935358 33674316

[42] Berlureau J, Anger R, Beaulieu E, Gourgues G, Bataille L, Jaubert J, Lartigue C, Sirand-Pugnet P, Arfi Y. 2026. An atypical F-type ATPase is necessary for the function of the antibody cleavage system MIB-MIP in mycoplasmas. PLoS Pathog 22:e1014043. doi:10.1371/journal.ppat.1014043 PMC12994834 41811866

[43] Wang J, Chitsaz F, Derbyshire MK, Gonzales NR, Gwadz M, Lu S, Marchler GH, Song JS, Thanki N, Yamashita RA, Yang M, Zhang D, Zheng C, Lanczycki CJ, Marchler-Bauer A. 2023. The conserved domain database in 2023. Nucleic Acids Res 51:D384–D388. doi:10.1093/nar/gkac1096 PMC9825596 36477806

[44] Marchler-Bauer A, Bo Y, Han L, He J, Lanczycki CJ, Lu S, Chitsaz F, Derbyshire MK, Geer RC, Gonzales NR, Gwadz M, Hurwitz DI, Lu F, Marchler GH, Song JS, Thanki N, Wang Z, Yamashita RA, Zhang D, Zheng C, Geer LY, Bryant SH. 2017. CDD/SPARCLE: functional classification of proteins via subfamily domain architectures. Nucleic Acids Res 45:D200–D203. doi:10.1093/nar/gkw1129 PMC5210587 27899674

[45] Kelley LA, Mezulis S, Yates CM, Wass MN, Sternberg MJE. 2015. The Phyre2 web portal for protein modeling, prediction and analysis. Nat Protoc 10:845–858. doi:10.1038/nprot.2015.053 PMC5298202 25950237

[46] Hildebrand A, Remmert M, Biegert A, Söding J. 2009. Fast and accurate automatic structure prediction with HHpred. Proteins 77 Suppl 9:128–132. doi:10.1002/prot.22499 19626712

[47] Gasteiger E, Hoogland C, Gattiker A, Se D, Wilkins MR, Appel RD, Bairoch A. 2005. Protein identification and analysis tools on the ExPASy server, p 571–607. *In* Walker JM (ed), The Proteomics Protocols Handbook. Springer.

[48] Sayers EW, Bolton EE, Brister JR, Canese K, Chan J, Comeau DC, Connor R, Funk K, Kelly C, Kim S, Madej T, Marchler-Bauer A, Lanczycki C, Lathrop S, Lu Z, Thibaud-Nissen F, Murphy T, Phan L, Skripchenko Y, Tse T, Wang J, Williams R, Trawick BW, Pruitt KD, Sherry ST. 2022. Database resources of the national center for biotechnology information. Nucleic Acids Res 50:D20–D26. doi:10.1093/nar/gkab1112 PMC8728269 34850941

[49] Kanci Condello A, Wawegama NK, Ekanayake D, Zhu L, Tivendale KA, Shil PK, Daly J, Mohotti S, Todhunter P, Underwood GJ, Noormohammadi AH, Markham PF, Browning GF. 2024. Evaluation of the safety and efficacy of the novel *Mycoplasma gallisepticum* vaccine, vaxsafe MG304, after spray-vaccination of 1-day-old specific pathogen-free chicks. Vet Microbiol 293:110093. doi:10.1016/j.vetmic.2024.110093 38692193

[50] Mahdizadeh S, Masukagami Y, Tseng C-W, Markham PF, De Souza DP, Nijagal B, Tull D, Tatarczuch L, Browning GF, Sansom FM. 2021. A *Mycoplasma gallisepticum* glycerol ABC transporter involved in pathogenicity. Appl Environ Microbiol 87:e03112-20. doi:10.1128/AEM.03112-20 PMC8208142 33741628

