# Supplementary material for: Interactions between immunoglobulin evasion systems in Mycoplasma gallisepticum and their impact on antibody-dependent complement-mediated killing
Source: Infect Immun. 2026 Oct 12;94(11):e00373-26. doi: 10.1128/iai.00373-26 (PMC13750126; doi:10.1128/iai.00373-26)
Supplement: Supplementary file 1 [file iai.00373-26-s0001.docx]

**FIG S1.** Western blot of purified recombinant Protein-M and CysP of *M. gallisepticum* probed with rabbit anti-GST antibody. Recombinant GST-Protein-M had the expected molecular weight of ~85 kDa and GST-CysP the expected molecular weight of ~80 kDa.

**FIG S2.** The cross-species activity of recombinant (A) Protein-M and (B) CysP of *M. gallisepticum*. Purified GST-Protein-M and GST-CysP were incubated with diluted sera from other avian (turkey, pheasant, and duck) and mammalian species (goat, sheep, rabbit, horse, pig and tammar wallaby). GST-Protein-M and proteins bound to it were purified by glutathione-affinity chromatography. The proteins were separated by 10% SDS-PAGE. Recombinant GST-Protein-M alone, GST-CysP alone and serum alone were included as controls. Green arrowheads, ~75 kDa avian IgA and IgM heavy chains; pink arrowheads, ~65 kDa IgY heavy chains; purple arrowheads, ~65–70 kDa mammalian IgM heavy chains; red arrowhead, ~40–45 kDa cleaved IgY heavy chain; dark blue arrowheads, ~50–55 kDa mammalian IgA and IgG heavy chains; light blue arrowheads, ~25 kDa immunoglobulin light chains.

**FIG S3.** Effect of immunoglobulin-binding and immunoglobulin-protease proteins on immunoglobulin-dependent complement-mediated killing of *M. gallisepticum*. Recombinant GST-MIB3940, GST-MIP3660, GST-Protein-M, and GST-CysP were tested individually or in combination for their capacity to influence the rate of complement-mediated killing of *M. gallisepticum* cells in the presence of rabbit antibodies specific for *M. gallisepticum*. Guinea pig serum served as the source of complement. The concentrations of viable *M. gallisepticum* at 0, 2, 4, 6, and 12 h were determined using the most probable number method and plotted as log_10_ colour changing units (CCU)/mL. RS, rabbit serum; C, complement; Protein-M, recombinant GST-Protein-M; CysP, recombinant GST-CysP; MIB, recombinant GST-MIB3940; MIP, recombinant GST-MIP3660; GST, recombinant GST tag protein.
