# Supplementary material for: Interactions between immunoglobulin evasion systems in Mycoplasma gallisepticum and their impact on antibody-dependent complement-mediated killing
Source: Infect Immun. 2026 Oct 12;94(11):e00373-26. doi: 10.1128/iai.00373-26 (PMC13750126; doi:10.1128/iai.00373-26)
Supplement: Supplementary file 2 [file iai.00373-26-s0002.docx]

| **Species/strain** | **GenBank protein accession no.** | **Gene locus tag/ORF^a^** | **Protein length**  **(aa)^b^** | **Molecular weight**  **(kDa)^b^** | **Protein homology^c^** | |
| --- | --- | --- | --- | --- | --- | --- |
|  |  |  |  |  | **Similarity (%)** | **Identity (%)** |
| ***M. gallisepticum* strain Ap3AS** | UQZ95710.1 | F9C22_03005 | 516 | 57.5 | 100 | 100 |
| ***M. gallisepticum* strain R_low_** | AAP56594.1 | MGA_1071 | 512 | 57.4 | 98 | 97 |
| ***M. gallisepticum* strain R_high_** | ADC30443.1 | MGAH_1071 | 506 | 56.8 | 97 | 96 |
| ***M. gallisepticum* strain S6** | AHB99548.1 | GCW_01420 | 507 | 56.8 | 96 | 95 |
| ***M. gallisepticum* strain F** | ADC31521.1 | MGF_4275 | 512 | 57.2 | 97 | 96 |
| ***M. gallisepticum* strain ts-11** | WP_065165599.1 | F6J64_RS01405 | 504 | 56.4 | 97 | 96 |
| ***M. imitans*** | WP_027121950.1 | P690_RS0101040 | 507 | 57.2 | 78 | 64 |
| ***M. tullyi*** | WP_182079009.1 | H3143_RS01205 | 517 | 58.1 | 84 | 73 |
| ***M. bradburyae*** | WP_255034880.1 | NMG68_RS01220 | 518 | 59.9 | 68 | 51 |
| ***M. iowae*** | WP_004025288.1 | EER00_RS03070 | 497 | 54.9 | 41 | 23 |
| ***M. pneumoniae*** | WP_010874756.1 | MPN_RS02245 | 582 | 65.9 | 38 | 24 |
| ***M. genitalium*** | WP_338427464 | MG_281 | 556 | 62.1 | 39 | 23 |
| ***M. penetrans*** | WP_011076965.1 | MYPE_RS00730 | 503 | 55.1 | 41 | 25 |

**S1 TABLE.** Protein-M homologs of *M. gallisepticum* and its orthologues in other mycoplasmas

^a^ Homologs were identified using BLASTX and the sequences retrieved from the NCBI database

^b^Computed using ProtParam (Expasy)

^c^Similarity and identity to the sequence of Protein-M of *M. gallisepticum* strain Ap3AS were calculated based on a pairwise Needleman-Wunsch Global Alignment of the protein sequences

| **MIB homologs** | **Protein-M homologue-F9C22_03005** | |
| --- | --- | --- |
|  | **Similarity (%)** | **Identity (%)** |
| F9C22_03940 | 29 | 15 |
| F9C22_03935 | 29 | 18 |
| F9C22_03930 | 32 | 20 |
| F9C22_03910 | 30 | 18 |
| F9C22_03850 | 31 | 18 |

**S2 TABLE.** Sequence similarity between the Protein-M homologue and the MIB proteins of *M. gallisepticum* strain Ap3AS

Similarity and identity with the Protein-M homologue were calculated based on a pairwise Needleman-Wunsch Global Alignment of the protein sequences

| **Species/strain** | **GenBank protein accession no.** | **Gene locus tag/ORF^a^** | **Protein length**  **(aa)^b^** | **Molecular weight**  **(kDa)^b^** | **Protein homology^c^** | |
| --- | --- | --- | --- | --- | --- | --- |
|  |  |  |  |  | **Similarity (%)** | **Identity (%)** |
| ***M. gallisepticum* strain Ap3AS** | UQZ95669.1 | F9C22_02750 | 564 | 63.4 | 100 | 100 |
| ***M. gallisepticum* strain R_low_** | AAP56642.2 | MGA_1153 | 564 | 63.4 | 100 | 99 |
| ***M. gallisepticum* strain R_high_** | ADC30490.1 | MGAH_1153 | 564 | 63.4 | 100 | 99 |
| ***M. gallisepticum* strain S6** | AHB99584.1 | GCW_01680 | 564 | 63.4 | 99 | 98 |
| ***M. gallisepticum* strain F** | ADC31472.1 | MGF_3970 | 564 | 63.4 | 99 | 99 |
| ***M. gallisepticum* strain ts-11** | WP_065164947.1 | F6J64_RS01670 | 564 | 63.4 | 100 | 99 |
| ***M. synoviae* 53** | AAZ43997.2 | MS53_0590 | 573 | 64.5 | 90 | 89 |
| ***M. pullorum*** | WP_235631831.1 | BLA55_RS04420 | 886 | 98.5 | 57 | 51 |
| ***M. gallinarum*** | WP_244874346.1 | Q346_RS03850 | 770 | 85.8 | 64 | 58 |
| ***M. glycophilum*** | VEU70495.1 | NCTC10194_00468 | 743 | 82.7 | 67 | 62 |
| ***M. gallinaceum*** | WP_223211649.1 | EXC51_RS04215 | 808 | 89.5 | 62 | 55 |
| ***M. meleagridis*** | WP_046096799.1 | EXC33_RS02155 | 668 | 75.0 | 47 | 32 |
| ***M. gallopavonis*** | VEU73033.1 | NCTC10186_00522 | 738 | 82.8 | 43 | 28 |

**S3 TABLE.** CysP homologs of *M. gallisepticum* and their orthologues in other mycoplasmas

^a^Homologs were identified using BLASTX and the sequences retrieved from the NCBI database

^b^Computed using ProtParam (Expasy)

^c^Similarity and identity percentages were calculated based on pairwise Needleman-Wunsch Global Alignment of protein sequences

| **MIB homologs** | **CysP homologue-F9C22_02750** | |
| --- | --- | --- |
|  | **Similarity (%)** | **Identity (%)** |
| F9C22_03920 | 28 | 16 |
| F9C22_03865 | 28 | 16 |
| F9C22_03660 | 28 | 15 |

**S4 TABLE.** Protein homology between CysP homologue and MIP proteins of *M. gallisepticum* Ap3AS

Similarity and identity to CysP were calculated based on a pairwise Needleman-Wunsch Global Alignment of protein sequences
